# Social attraction mediates collective foraging decisions in invasive yellow-legged hornets

**DOI:** 10.1093/beheco/arag126

**Published:** 2026-10-27

**Authors:** Mathilde Lacombrade, Charlotte Doussot, Blandine Mahot-Castaing, Jacques Gautrais, Loïc Goulefert, Edgar Ferrieu, Amelie Bourg, Denis Thiéry, Fanny Vogelweith, Mathieu Lihoreau

**Affiliations:** Centre de Biologie Intégrative (CBI); CNRS, Université de Toulouse, 165 Avenue Grundberg-Manago, 31062 Toulouse, France; UMR 1065 Santé et Agroécologie du Vignoble, INRAE, 71 Avenue Edouard Bourlaux, 33883 Villenave d’Ornon, France; M2i Biocontrol, 370 route de Cauzenil, Parnac 46140, France; Centre de Biologie Intégrative (CBI); CNRS, Université de Toulouse, 165 Avenue Grundberg-Manago, 31062 Toulouse, France; Centre de Biologie Intégrative (CBI); CNRS, Université de Toulouse, 165 Avenue Grundberg-Manago, 31062 Toulouse, France; Centre de Biologie Intégrative (CBI); CNRS, Université de Toulouse, 165 Avenue Grundberg-Manago, 31062 Toulouse, France; Centre de Biologie Intégrative (CBI); CNRS, Université de Toulouse, 165 Avenue Grundberg-Manago, 31062 Toulouse, France; M2i Biocontrol, 370 route de Cauzenil, Parnac 46140, France; M2i Biocontrol, 370 route de Cauzenil, Parnac 46140, France; UMR 1065 Santé et Agroécologie du Vignoble, INRAE, 71 Avenue Edouard Bourlaux, 33883 Villenave d’Ornon, France; Entomo-Logik, 46000 Cahors, France; Centre de Biologie Intégrative (CBI); CNRS, Université de Toulouse, 165 Avenue Grundberg-Manago, 31062 Toulouse, France

**Keywords:** aggregation, collective behavior, group-size effect, social attraction

## Abstract

Group-living animals commonly use social information to locate and exploit resources. In many insects, fish, birds and mammals, this leads to collective foraging decisions by which animals aggregate on a single food source despite several alternatives of different or equal qualities. Here, we report collective foraging decisions in a widespread invasive hornet (*Vespa velutina nigrithorax*) that predates on bees and consume plant nectar. When given a choice between 2 identical sugar solutions (in feeders or in traps), wild hornets distributed asymmetrically between the 2 options, and this was more frequent as the number of hornets increased. Priming one of the food sources with dead hornets predictably biased the aggregation toward this particular food source, irrespective of whether the baits were conspecifics or hornets from a different species. Inter-attraction in yellow-legged hornets is thus passive and non-specific, possibly mediated by visual and/or chemical cues displayed by dead hornets. Social foraging may provide important benefits to hornets invading new territories and bring new perspectives for population control.

## Teaser

Yellow-legged hornets, also known as Asian hornets, are invasive insect predators across Europe, East Asia, and Northern America. Here we show how hornets in search for food use social cues to join other foraging hornets, often resulting in large, mixed-species, aggregations on sugar syrup feeders or in baited traps. We suggest this gregarious behavior is key to the ecological success of invasive populations and discuss how it could be used for biocontrol.

## Introduction

Social animals, such as insects, fish, birds, and mammals, often make collective decisions when it comes to fleeing danger, choosing a new home, or finding food (Sumpter 2010). These collective behaviors can involve dozens to billions of individuals (Sayin et al. 2025) and provide important fitness benefits through swarm intelligence (Couzin 2009; Krause et al. 2010; Sasaki et al. 2013). Strikingly, complex group behaviors often emerge from a limited number of generic principles, such as feedback loops and quorum sensing by which the behavior of some individuals in the group influences that of others in a non-linear manner (Camazine et al. 2001). For instance, when foraging for food, many ant species deposit pheromone trails that guide nestmates toward discovered sites. Newcomers that successfully found the food, further amplify the signal with more pheromone deposition, thereby acting as positive feedback. When a large number of ants are engaged in the task, the pheromone trail becomes so strong that the colony exploits one food source in particular among several alternatives (Detrain and Deneubourg 2008). If the different food sources vary in quality, the ants can collectively select the most profitable one (Jeanson et al. 2012). Conversely, food depletion and pheromone evaporation constitute negative feedbacks that can reduce or stop the expression of the collective behavior (Jeanson et al. 2012).

Collective decisions can be experimentally demonstrated by giving a group of animals a choice between 2 identical options, as for instance 2 food sites of equivalent size and nutritional value (Deneubourg and Goss 1989). If the animals use social cues to decide where to go, feedback loops induce asymmetrical distributions of the individuals on the 2 sites, with 1 site (ie winner site) attracting a significantly larger proportion of the populations than the other (Detrain and Deneubourg 2008). The strength of the collective decision, as measured by the degree of asymmetry between the 2 sites, typically increases with group size (ie the number of individuals involved in foraging) and the amount of social cues available (Lihoreau et al. 2010). Importantly, while several animals make collective decisions through active signaling (eg ant pheromone trail to food sources [Wilson 1962], honeybees waggle dance advertising feeding location [von Frisch 1967]), asymmetrical distributions can also emerge through a range of passively displayed social cues that can attract or retain individuals on site (eg body scents of cockroaches choosing a resting site [Amé et al. 2006]).

Here, we used this binary choice experimental approach in the field to explore the possibility for collective foraging decision-making in an ecologically and economically important social wasp, the yellow-legged hornet (*Vespa velutina nigrithorax*). In recent years, this hornet native of South Eastern Asia, has become invasive across Western Europe (Haxaire et al. 2006), Eastern Asia (Kim et al. 2006), and Northern America (Hoebeke et al. 2024). In all these areas, hornets collect flower nectar as a source of energy (Ueno 2015; Bouchebti et al. 2022) but also predate on invertebrates for proteins and lipids, causing strong concerns for the local biodiversity and beekeeping activities. Honeybees, in particular, account for 33% to 66% of yellow-legged hornets' diet (Villemant et al. 2011; Rome et al. 2021), leading many beekeepers to use handcrafted traps baited with sugary food to control populations (Rojas-Nossa et al. 2018). Using this approach to protect our own experimental apiaries, we observed that the same traps on the same site often caught very different numbers of hornets, suggesting the occurrence of social attraction between hornets. Social attraction on feeding sites has already been documented in wasp species of the genus Vespula, leading to density dependent aggregations (eg D’Adamo and Lozada 2005; Lozada et al. 2016). In the European wasp (*Vespula germanica*), social attraction is mediated by visual and chemical cues (D’Adamo et al. 2000; Buteler et al. 2017), and these cues can be used in the foraging decisions of closely related species exploiting the same resources (Raveret Richter and Tisch 1999).

To test for the existence of a social attraction in foraging yellow-legged hornets, we gave populations of wild hornets a choice between 2 identically attractive food sources placed next to each other (2 feeders or 2 traps containing sugar-rich solutions; Fig. 1) and quantified their distribution through time. If the hornets attracted each other, we expected them to distribute asymmetrically between the 2 food sources, and the frequency of significant distribution asymmetries should increase with group size.

**Figure 1:**
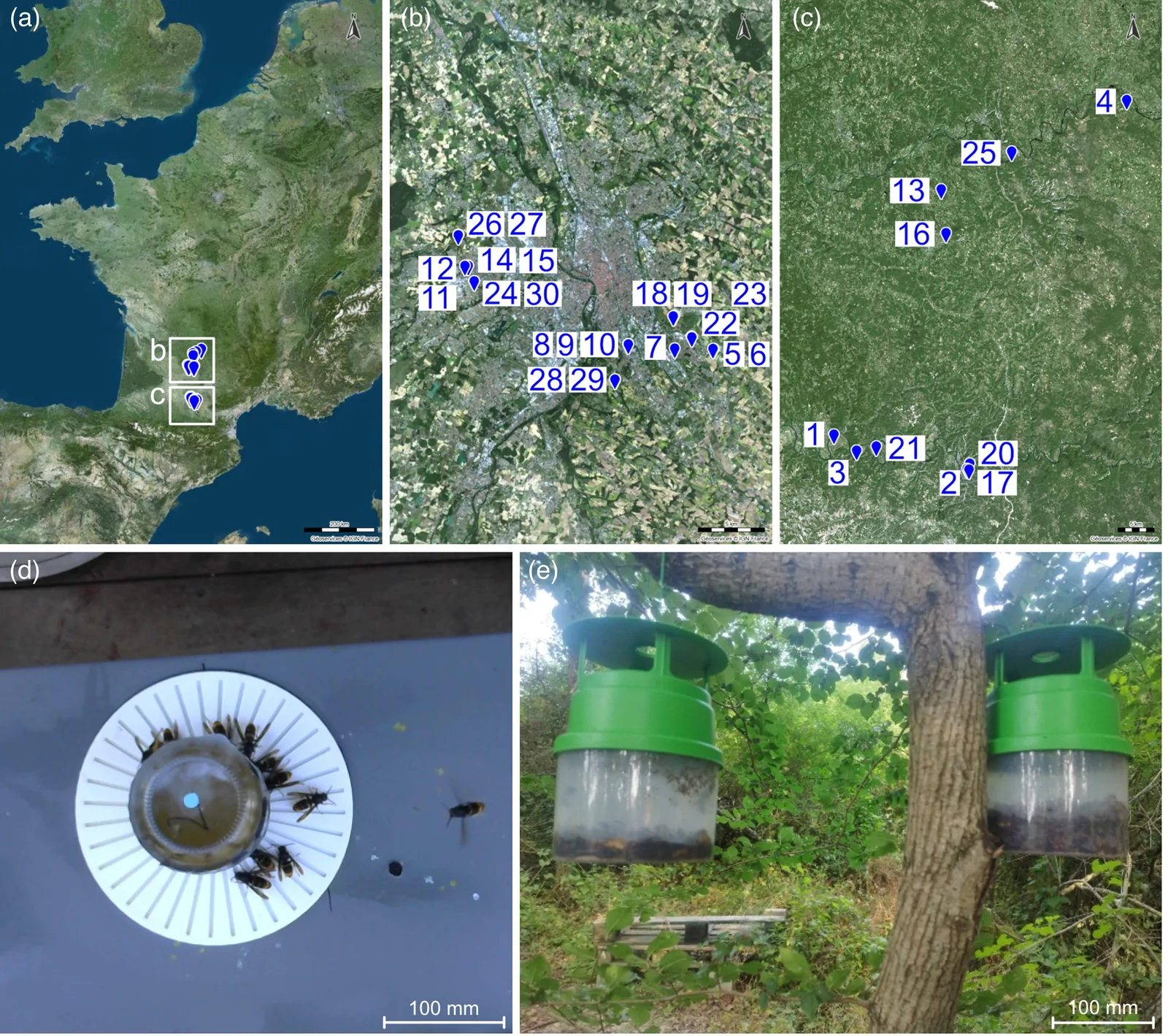
(a) Location of the 30 experimental sites (n°1–30 see details in Table S1; bottom right scale corresponds to 200 km) near (b) Toulouse (scale: 5 km) and (c) Cahors (scale: 5 km). Each site was equipped with 2 gravity feeders or 2 baited traps. The feeders (d) were made of a white horizontal plastic landing platform fitted with an opaque vertical black container that released diluted honey solution through gravity. The baited traps (e) consisted of a transparent plastic bowl (diameter: 136 mm; height: 193 mm; weight: 169 g) covered with an opaque green lid (diameter: 16 mm), containing wine, beer and sugar syrup. The traps were placed 50 cm apart, hanging from a tree branch. The maps were created with IGN, Open License Etalab 2.0.

## Materials and methods

### Experimental sites

We conducted the experiments in France over 2 field seasons (2022 and 2024). We used 30 sites located in 12 apiaries of comparable sizes (Fig. 1a to c, see Tables S1 and S2 for details about site assignment to each experiment). These sites hosted wild populations of invasive yellow-legged hornets (*V. velutina nigrithorax*, hereafter referred to as VV). The sites were also populated with native European hornets (*Vespa crabro*, hereafter referred to as VC) that occupy a similar ecological niche as VV. On each year, we made all the observations between June and November in order to exclusively test hornet workers (gynes and males are seen before and after this period) (Rome et al. 2015).

### Feeder choice experiments

#### Choice between 2 identical feeders

We tested inter-attraction in nectar foraging hornets by placing 2 identical feeders next to each other (hereafter feeder 1 or 2). In case of inter-attraction, we expected one feeder to be chosen by a majority of hornets. We also expected that the frequency of asymmetrical distributions of hornets between the 2 feeders would increase with the total number of hornets on both feeders at a given time (hereafter group size), and that the position of the feeder attracting the most individuals (left or right, hereafter position A or B) would vary across replicates.

For this experiment, we positioned 2 gravity feeders 50 cm apart on a 1 m high table (Fig. 1d). The feeders consisted in a glass jar (diameter: 5 cm, height: 10 cm) containing liquid food (1:3 ratio of commercial honey [wild flowers honey, Famille Michaud & Co, Gan] to water), placed upside down on top of a 3D printed plastic white landing platform (diameter: 20 cm, height: 0.5 cm) with radial trenches from which hornets could drink. We estimated that each landing platform was large enough to host a group of up to 265 hornets simultaneously. This calculation was made based on the total surface of the landing platform, assuming hornets were elliptical objects measuring 1.98 cm long and 0.57 cm wide (Sipos et al. 2025). Note that this is an underestimation as hornets could occasionally access food while landing on top of each other (personal observation). Each jar was covered with dark tape so that the foraging hornets could not see the volume of food available in the feeders upon making a choice. With this design, foraging hornets could choose a feeder solely based on olfactory, tactile and visual cues provided by other hornets already landed on the platform. During the observations, we swapped the positions of the 2 feeders every 15 min to make sure that attraction to a feeder was solely due to social cues, and not to the identity of the feeders (1 or 2) or to their spatial positions (A or B). The feeders remained at a given location for 30 consecutive minutes (15 min in position A and 15 min in position B). We ran these experiments in 5 different locations at one experimental site (site 8 in Table S1) during 4 days (Fig. 1b), totaling 30 replicates.

#### Data collection on feeders

For each replicate, we recorded the number of hornets on each feeder every 30 s using a camera (C270 HD Webcam, Logitech) placed 60 cm above each feeder. The 2 cameras were operated by a Python3 program (Python software foundation 2023) using *vidgear* library (Thakur et al. 2023) that automatically took pictures during 2 h (30 min × 4) between 1 PM and 6 PM (240 frames for 2 h, 1,920 frames in total). We used computer vision with Python's OpenCV library to quantify the number of darkened pixels (ie hornet) on the white surfaces of the feeders' landing platforms. This involved using adaptive binarization to convert specific luminosity thresholds into black (for hornets) and white (for the background and feeder). To isolate the counting to the feeder area, we applied circle detection tools available in OpenCV. To convert the number of darkened pixels into an estimate of the number of hornets, we used a calibration curve (a polynomial fit of the number of computer-retrieved black pixels throughout a sample of images where the number of hornets was counted by an experimenter (mean error ± SD: 6.99 ± 8.34). We denoted hereafter the total number of hornets in both feeders at a given time as “group size.”

#### Trap choice experiments

We tested whether the observations made on gravity feeders could be verified in another context, across more experimental sites and with a larger number of hornets, by giving hornets a choice between 2 baited traps.

In each experimental site, we placed 2 commercial hornet traps (Smart Trap, M2i Biocontrol) 50 cm apart (Figs. 1d and S1d). The 2 traps were hung on the same tree branch, between 1 and 3 m above ground. Each trap was made of a transparent bowl containing standard attractive bait (white wine 50% v/v, beer 25% and 25% sugared syrup) and covered with an opaque funnel-shaped lid with a roof. The lid enabled the insects to enter the trap but not to exit. With this design, the hornets could use both olfactory and visual social cues to choose a trap. The traps had low selectivity levels since we caught hornets of 2 species (ie VV and VC) and a also variety of other insects including Hymenoptera, Lepidoptera and Diptera (see details in the Results).

### Choice between 2 identical traps

We first tested for inter-attraction among hornets by placing 2 identical baited traps (traps 1 and 2) next to each other. If the foraging hornets were attracted by trapped hornets, we would expect 1 of the 2 traps to be chosen by most individuals. The frequency of significant asymmetries should vary with overall group size (number of hornets in both traps), and the position of the trap attracting the majority of hornets (A or B) should vary across replicates.

#### Choice between an empty trap and a trap primed with dead hornets of the same species

We then tested whether the social cues involved in inter-attraction were displayed by dead hornets or whether the hornets already present in the traps needed to be alive. Here hornets were given a choice between an empty trap (containing no hornet) and a primed trap (containing 15 dead VV hornets randomly sampled in dedicated traps collected every day and cleaned with fresh water before the experiment). If the dead hornets displayed attractive social cues, we would expect the primed trap to be more frequently chosen by most individuals.

#### Choice between an empty trap and a trap primed with dead hornets of another species

In this final experiment we tested whether the social cues involved in inter-attraction were specific or not. Hornets were given a choice between an empty trap (containing no hornet) and a primed trap (containing 15 dead VC hornets randomly sampled in dedicated traps collected every day and cleaned with 70% alcohol before the experiment). If the inter-attraction by VV hornets was specific, we would expect one of the traps to be chosen randomly by the majority of individuals, irrespective of the presence of dead VC hornets in the traps.

#### Data collection in traps

We counted the number of hornets in the traps between 3 and 14 days after setting the traps on site (Tables S1 and S2). We voluntarily varied this duration in order to maximize variance in the total amount of hornets trapped in our dataset, ie group size (range: 1 to 422 individuals). For each record, we emptied the 2 traps simultaneously and counted the total number of VV and VC hornets in each trap. We also estimated the amounts of other insects caught in the traps as a percentage of the bowl occupied in order to evaluate their potential influence on hornet attraction.

### Statistical analysis

We performed all the statistical analyses with R 4.5.0 (R Core Team 2025). The raw data are available in a github repository (https://github.com/lacmathilde/Raw_data.git).

For all experiments, at each location of each site, we considered the feeder or trap that attracted the largest number of hornets on each observation period as the “winner” option.

In the choice experiment between 2 identical feeders, we tested inter-attraction by running a proportion test on each count. We first evaluated if the proportion of hornets on the winner feeder was significantly different from random (ie 50%) using the function binom.test (*stats* package). We then ran a test to assess whether hornets had accumulated on one of the feeders in a non-random way considering all the counts using the function prop.test. Finally, we assessed the influence of group size on the frequency of asymmetric distributions between 2 identical feeders by performing a generalized linear mixed-effects model with a binomial distribution using glmer function (lme4 package [Bates et al. 2015]). We used the same statistical analyses for the choice experiment between 2 identical traps.

In the choice experiment between an empty trap and a primed trap, we tested the influence of dead hornets on captures. We evaluated whether the primed trap was more attractive to hornets and to what extent it became more frequently the winner trap. We then tested if the proportion of captures across the winner and primed traps was different from 0.5 by using a proportion test on each count.

In all trap choice experiments, we evaluated the specificity of social attraction by calculating the proportion of VV and VC hornets caught in the same winner trap. To test whether other insects (Vespidae, Apidae, Lepidoptera, and Diptera) (Rome et al. 2015) also caught in the traps influenced the observed distributions of hornets (because they would predate on them), we performed a linear mixed effect model (LMM) with Poisson distribution using the function lmer (*lme4* package) using *the percentage of trap occupied by the mass of captured insects* as fixed factors and *site* as random factor. We then ran an ANOVA on this model using the function Anova (*car* package [Fox and Weisberg 2018]).

## Results

### Choice between 2 identical feeders

We tested for the existence of an inter-attraction between foraging hornets by giving wild hornets a choice between 2 feeders containing ad libitum diluted honey. Over the 16 h of observations, we exclusively observed VV hornets (no count of VC on feeders) and the group size (total number of hornets landed on the 2 feeders at a given time) varied between 0 and 215 individuals.

The frequency of asymmetric distributions between the 2 feeders at a given location increased significantly with group size (Fig. 2; GLM with binomial distribution, effect of *group size* on *asymmetry*: df = 934, *z* = 5.365, *P* < 0.001). For small groups of <50 hornets, asymmetric distributions were observed in 36.32% of the cases (93 out of 256 measures). This percentage remained low in groups of 50 to 99 hornets with 22.48% of asymmetric distributions (29 out of 129 measures), but increased in large groups of 100 to 149 hornets to 67.53% (52 out of 77 measures), and reached a maximum in very large groups of 150 to 199 hornets with 87.50% (7 out of 8) (Proportion test: χ^2^ = 19.97, df = 3, *P* < 0.001; see pairwise comparisons in Table S3). This group size effect on the frequency of asymmetrical distributions is the signature of a collective decision driven by inter-attraction of hornets on feeders.

**Figure 2:**
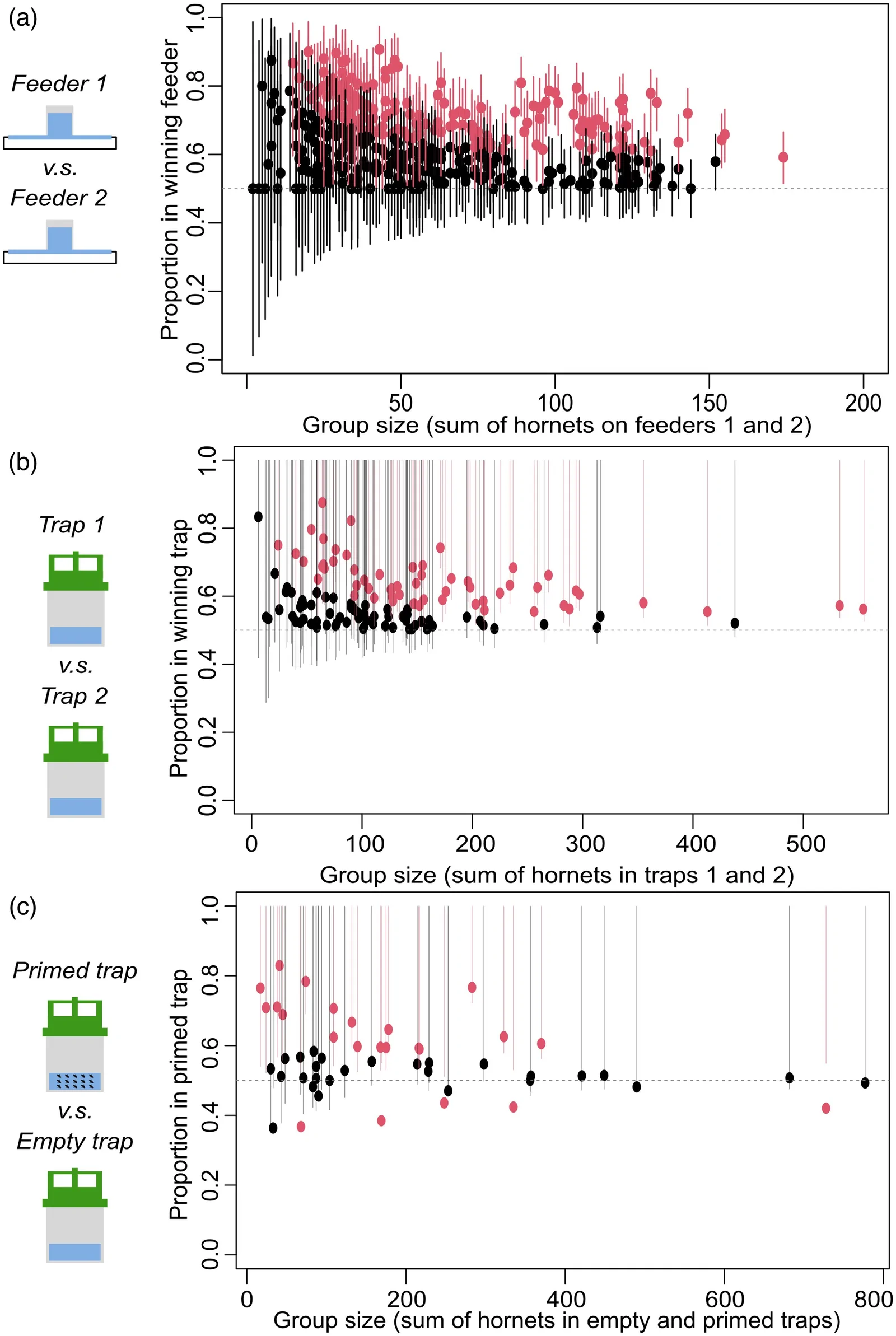
Proportions of hornets in (a) the winner feeder, (b) the winner trap, and (c) the primed trap according to group size. Red dots represent statistically significant asymmetric distributions between the 2 options (proportional test, *P* < 0.05). Black dots are non-significant asymmetries. Solid lines are 95% CIs. Dashed gray lines indicate random distributions.

Interestingly, when the distributions of hornets were significantly asymmetric, we observed frequent changes in the identity of the winner feeder through time (Fig. 3). Overall, feeder 1 was the winner feeder in only 52.46% of the records (181 out of 345 measures), which means that the hornets did not have any persistent preference for 1 of the 2 feeders (binomial test, *P* = 0.39). This temporal alternance in the identity of the winner feeder indicates that inter-attraction was not mediated by long-lasting cues left by hornets on the feeders, such as chemical traces, but rather suggests the implication of visual cues or short-range odors carried by the hornets themselves.

**Figure 3:**
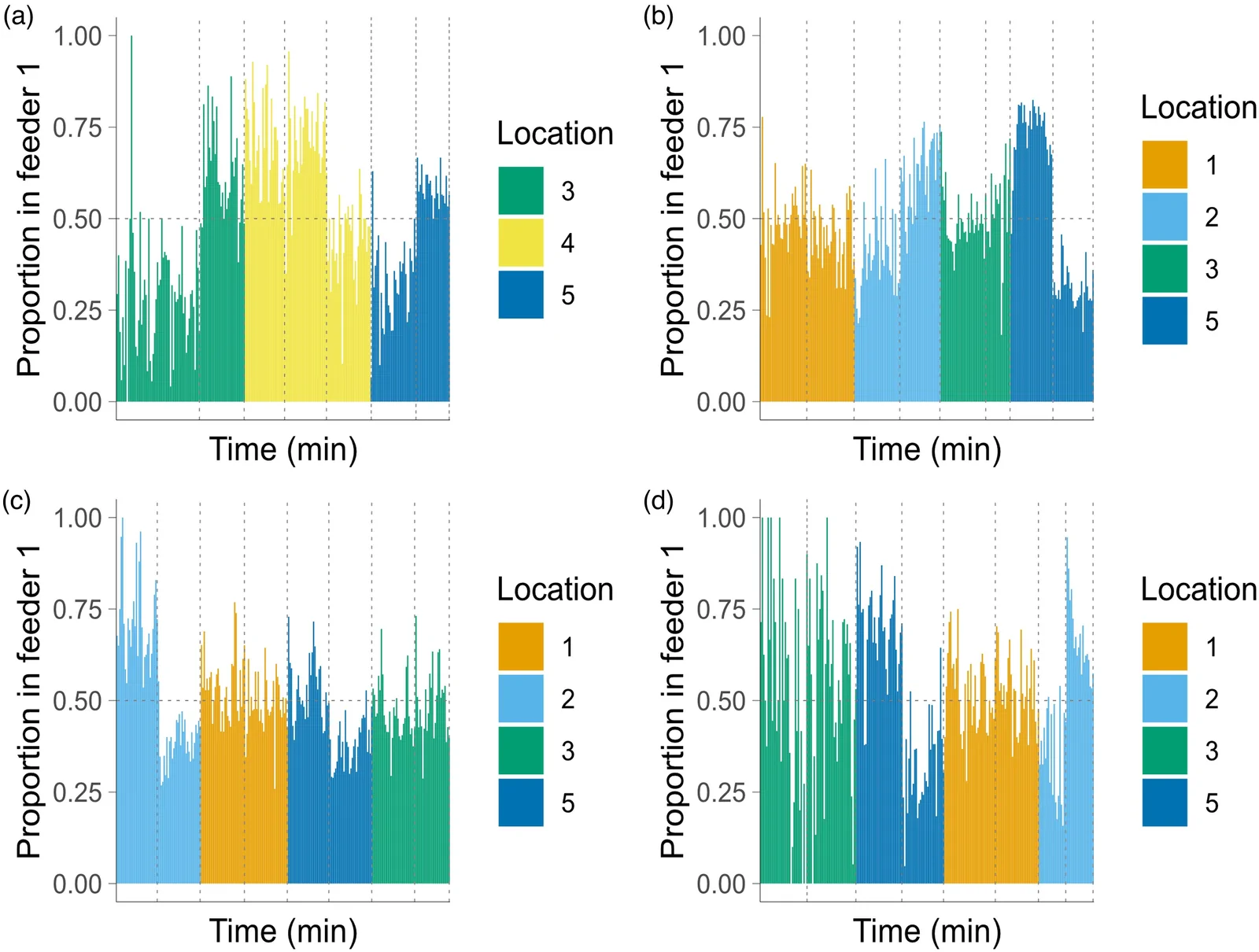
Proportion of hornets on feeder 1 across experimental day (time) (a: day 1, b: day 2, c: day 3, d: day 4) and in different locations (orange: location 1, light blue: location 2, green: location 3, yellow: location 4, dark blue: location 5) of experimental site 8. The horizontal dashed gray line represents random distribution. When more than half of the hornet population is located on feeder 1, it becomes the winner feeder (feeder 1 wins when the proportion of hornets on feeder 1 > 0.5, feeder 2 wins when the proportion of hornets on feeder 1 < 0.5). The vertical dashed black lines represent the changes of feeder positions (A or B) every 15 min.

### Choice between 2 identical traps

We then investigated whether the collective decisions observed for feeders could also be observed for baited traps. This approach enabled us to test more individuals and in more sites.

In this approach, we caught hornets of the 2 species: VV and VC. When considering both species together, the frequency of asymmetrical distributions of hornets among traps increased significantly with the total number of hornets in the 2 traps (group size) (GLM with binomial distribution, effect of *group size* on *asymmetry*: df = 123, *z* = 3.12, *P* < 0.001). The number of records in which the proportion of hornets in the winner trap was significantly higher than that in the looser trap increased with group size irrespective of the site, from 31.48% (17 out of 54 records) in small groups with <100 hornets in both traps, to 55.56% (40 out of 72 records) in large groups of 100 or more hornets in both traps (proportional test: χ^2^ = 6.28, df = 1, *P* = 0.01). This group size effect was comparable to that reported above with the feeders (Proportion test, group size <100: χ^2^ = 0.002, df = 1, *P* = 0.96, group size ≥100: χ^2^ = 1.610, df = 1, *P* = 0.205).

If inter-attraction were specific, the asymmetrical distributions of VV should be independent of the distribution of VC in the traps. However, when considering the 2 species separately, we found that in 68.25% of the cases (86 out of 126 records), the winner trap was the same for VV and VC (Fig. 2). The lack of specificity was further supported by the fact that traps also caught a diversity of other non-hornet insects, and this number was positively correlated to the total number of hornets of both species in the traps (LMM, percentage of insect mass: χ^2^ = 172.1, df = 1, *P* < 0.001).

### Choice between an empty trap and a trap primed with hornets

To further explore the nature of the attraction cues driving the collective decisions of hornets, we primed one trap with 15 dead hornets and compared the number of hornets caught in the 2 traps on the same site. If dead hornets displayed attraction cues, the primed trap should become the winner trap more often than expected by chance.

When considering hornets from the 2 species altogether, the primed trap was the winner trap in 82.35% of the cases (42 out of 51 records; Fig. 2). This confirms that the choice of a trap is influenced by the presence of hornets already caught inside, be they dead or alive.

If attractive cues were specific, we would expect traps baited with one species to be the winner trap only for that particular species. Accordingly, the VV primed trap became the winner trap for VV in 100% of the cases (39 out of 39 records; binomial test: *P* < 0.001, confidence interval, CI: [0.91; 1.00]). However, and in accordance with the previous experiment, the VV primed trap also became the winner trap for VC overall but in only 67.85% of the cases (19 out of 28 records; binomial test: *P* = 0.09, CI: [0.47; 0.84]; 5 records showed equal distribution between the primed and the empty traps). Similarly, the VC primed trap became the winner trap for VC in 100% of the cases (9 out of 9 records; binomial test: *P* = 0.004, CI: [0.66; 1.00]) and the winner trap for VV in 66.66% of the cases (6 out of 9 records; binomial test: *P* = 0.50, CI: [0.30; 0.93]).

Without distinguishing between hornet species, the efficiency of the traps baited with either VV or VC was comparable (χ^2^ test, χ^2^ = 0.001, df = 1, *P* = 0.924). Indeed, primed traps became winner traps in 84.61% of the cases (33 out of 39 records) when primed with *VV* and 75.00% of the cases (9 out of 12) when primed with *VC*. Although the inter-attraction appears to be stronger within the same species, both species were attracted to each other.

## Discussion

Collective decision-making is a hallmark of animal sociality (Sumpter 2010). Here, using binary choice experiment designs in the field, we report collective foraging decisions for food resources in wild populations of the invasive yellow-legged hornet *V. velutina nigrithorax*. The gregarious behavior of hornets on feeders and in traps is density dependent, and can involve hornets of different species, suggesting that collective decision-making results from inter-attraction mediated by non-specific cues passively displayed by foraging hornets. We discuss our results in the light of previous observations in other social wasps.

Our observation of density-dependent asymmetrical distributions of hornets given a choice between 2 identical food sources is a strong signature of collective decisions mediated by inter-attraction (Lihoreau et al. 2010, 2016; Jeanson et al. 2012). Significant asymmetries on feeders containing sugar solutions more than doubled in frequency with group size, from 36.32% when the number of individuals was below 50 to 87.50% when this number reached 150 or more. Critically, a similar density-dependent collective response was observed in hornets facing 2 identical traps also baited with sugar solution, suggesting that the same inter-attraction mechanism is involved in the 2 aggregation processes. While our results do not enable to identify potential critical densities above which collective decisions systematically emerge (ie quorum), using primed traps show that 15 dead hornets are sufficient to bias the hornet distributions toward the primed trap in ca. 80% of the cases, irrespective of the hornet species used as attractor. These observations in hornets echo those of density dependent-aggregations on food resources previously reported in some other social wasp species, such as European wasp *V. germanica* (D’Adamo and Lozada 2005) and the camoati *Polybia occidentalis* (Hrncir et al. 2007).

In contrast to the well-documented collective decisions of ants, bees and some other social insects in which individuals attract their nestmates on site using active recruitment behaviors (Wilson 1962; von Frisch 1967), hornets did not display evident active signaling through specific social interactions around food resources or through long-lasting scent marking of feeders, as revealed by frequent alternances in the identity of winner feeders during the course of the observations. Inter-attraction was rather likely mediated by short-range cues passively displayed by hornets on feeders and in traps. Assuming the same mechanism was at play in all our experiments, the fact that hornets in search for food could not interact with trapped insects before actually entering the trap, rules out a potential implication of tactile cues during physical contacts between foraging hornets. The social cues may therefore be of chemical and/or visual nature. In the European wasp *V. gemanica*, local attraction to feeding sites is predominantly mediated by volatile chemical cues emitted by nestmates, but vision also plays a role (D’Adamo et al. 2000, 2001; Buteler et al. 2017). Previous observations also suggest that successful wasp foragers recruit conspecifics during social interactions in the nest, although the precise nature of these interactions remain to be identified (Lozada et al. 2016).

In our experiments, since baits primed with dead yellow-legged hornets successfully biased the collective choices, as well as did dead European hornets but to a lesser extent, the attractive cues are not entirely specific and may be used by other species exploiting similar food sources, just like insect pollinators such as bees use social information from various species in order to locate most profitable nectar sources (Dawson and Chittka 2012). Interestingly, *Vespula* wasps also appear to use heterospecific cues to choose feeders, although the effects of social cues on the behavioral responses of wasps (attraction, repulsion) strongly vary across species (Raveret Richter and Tisch 1999; Pereira et al. 2016).

At this stage we cannot definitely exclude the possibility that the accumulation of hornets on feeders and in traps was mediated by different processes and cues. For instance, hornets may have been attracted in the traps by cues indicating the presence of potential preys (although we are not aware of any report of intraspecific necrophagy in these hornets), whereas the feeders were exclusively visited by yellow-legged hornet conspecifics. Future experiments are thus needed to further explore the nature of these attractive cues, for instance by manipulating the presence of odors and visual information available on feeders and in traps.

Irrespective of the mechanisms mediating inter-attraction, collective foraging in hornets likely provides important benefits for resource localization and exploitation, through information transfer and swarm intelligence, as described in other social animals (Nicolis and Dussutour 2008). First, the fact that yellow-legged hornets were observed to use social information provided by another hornet species may constitute an advantage regarding food localization. For instance, generalist bees, such as bumblebees and honey bees, are well known to use social information from other pollinators to learn about plant species and foraging techniques to obtain the best nectar and pollen resources in their shared habitats (Chittka 2022). In Western Europe, yellow-legged hornets and European hornets are often seen foraging at in the same areas at similar times of the day (Monceau et al. 2015; Cini et al. 2018). They may thus mutually benefit from the presence of each other in terms of information gain regarding plant choice for nectar collection. More generally, collective foraging could enable hornets to adapt to a wide range of habitats by capitalizing on signals already present in their surroundings, ultimately leading to different species benefiting from the presence of each other through heterospecific collective behavior, as previously documented in other ecological associations (Shridar et al. 2009; Schmitt et al. 2014; Boulay et al. 2016; Lukas et al. 2023). Sharing foraging information within and between species may further explain their ecological success and rapid spread in newly colonized areas allowing them for enhanced tracking of most profitable resources in their environment, increased foraging efficiency and higher adaptability to complex environments (Chapman and Bourke 2001; Avarguès-Weber et al. 2013; Boulay et al. 2016). Whether collective foraging dynamics can also be observed in hornets hunting for preys is an open question that will deserve further investigation, possibly using a similar approach of binary choice assays.

Our observation brings new fundamental knowledge about the foraging ecology of hornets. It also provides new interesting insights in the context of population control in a major invasive species worldwide. Over the past decades, research on European wasps invasive in Northern, Central and South America, South Africa and Australia has enabled considerable advances in trap design and population control (Lester and Beggs 2019). Similarly, in the case of yellow-legged hornets, our findings raise the possibility of exploiting the inter-attraction underpinning collective hornet decisions to increase the efficiency of traps by baiting existing trap designs with dead hornets or lures displaying key attractive cues. While many beekeepers already implement this practice based on empirical observations, our data now provide scientific evidence for this phenomenon and offer quantitative predictions related to the effect of group size.

## Supplementary Material

arag126_Supplementary_Data

## Data Availability

Analyses reported in this article can be reproduced using the data provided by Lacombrade et al. (2026).
