# Supplementary material for: Social attraction mediates collective foraging decisions in invasive yellow-legged hornets
Source: Behav Ecol. 2026 Oct 27;37(6):arag126. doi: 10.1093/beheco/arag126 (PMC13711668; doi:10.1093/beheco/arag126)
Supplement: Supplementary file 1 [file arag126_supplementary_data.docx]

**Social attraction mediates collective foraging decisions in invasive yellow-legged hornets**

Mathilde Lacombrade^1,2,3,†^, Charlotte Doussot^1^, Blandine Mahot-Castaing^1^, Jacques Gautrais^1^, Loïc Goulefert^1^, Edgar Ferrieu^3^, Amelie Bourg^3^, Denis Thiéry^2^, Fanny Vogelweith^4,^*, Mathieu Lihoreau^1,^*^,†^

^1^ Centre de Recherches sur la Cognition Animale (CRCA), Centre de Biologie Intégrative (CBI), Université de Toulouse ; CNRS, UPS, France

^2^ UMR 1065 Santé et Agroécologie du Vignoble, INRAe, F-33883 Villenave d’Ornon, France

^3^ M2i Biocontrol, 370 route de Cauzenil, 46140 Parnac, France

^4^ Entomo-Logik, 46000 Cahors, France

*****These authors contributed equally to the work

^†^Corresponding authors: lacombrademathilde@gmail.com; mathieu.lihoreau@utoulouse.fr

**Supplementary materials**

Table S1: Summary table of each counting information to identify an inter-attraction between hornets (two identical traps)

Table S2: Summary table of each counting information to identify the specificity of the inter-attraction between hornets (one primed trap and one emptied trap)

Table S3: Statistical results from pairwise comparison tests with a Bonferroni correction between the proportion of significant asymmetry of different hornets’ group size (designated as GS XX-XX hornets).

|  | **GS 0-49** | **GS 50-99** | **GS 100-149** |
| --- | --- | --- | --- |
| **GS 50-99** | X²= 5.40, df=1,p=0.13 | - | - |
| **GS 100-149** | X²= 23.2, df=1, p<0.001 | X²= 36.80, df=1, p<0.001 | - |
| **GS 150-199** | X²= 5.13, df=1, p=0.14 | X²=9.90, df=1,p=0.001 | X²=0.27, df=1, p=1.00 |
